# Effects of High-Intensity Interval Training and Moderate-Intensity Training on Stress, Depression, Anxiety, and Resilience in Healthy Adults During Coronavirus Disease 2019 Confinement: A Randomized Controlled Trial

**DOI:** 10.3389/fpsyg.2021.643069

**Published:** 2021-02-24

**Authors:** Yolanda Borrega-Mouquinho, Jesús Sánchez-Gómez, Juan Pedro Fuentes-García, Daniel Collado-Mateo, Santos Villafaina

**Affiliations:** ^1^Faculty of Sport Science, University of Extremadura, Cáceres, Spain; ^2^Centre for Sport Studies, Rey Juan Carlos University, Madrid, Spain

**Keywords:** confinement, COVID-19, HIIT, MIT, mental health

## Abstract

**Objective:** This study aimed to compare the effects of two intervention programs, (1) high-intensity interval training (HIIT) and (2) moderate-intensity training (MIT), on anxiety, depression, stress, and resilience during the confinement caused by the coronavirus disease 2019 (COVID-19) in healthy adults.

**Methods:** A total of 67 adults who participated were randomly assigned to two groups: HIIT and MIT groups. The MIT group had to perform a home-based intervention based on aerobic exercises, whereas the HIIT group had to perform a home-based intervention based on HIIT exercises. The two groups (HIIT and MIT) had to complete the same physical exercise volume, 40 min per session (6 days per week) during the confinement period (6 weeks). Depression, anxiety, stress, and resilience were assessed before and after the intervention.

**Results:** Results showed that HIIT and MIT significantly reduced the stress, anxiety, and depression as well as increase the resilience (*p* < 0.05). Moreover, the improvements obtained in the HIIT group seem to be greater than those of the MIT group in depression (*p* < 0.05).

**Conclusions:** HIIT and MIT decreased anxiety, stress, and depression as well as increased resilience during the COVID-19 confinement. In addition, the HIIT intervention seemed to be more beneficial to reduce depression than the MIT intervention.

## Introduction

In December 2019, a series of pneumonia cases with unknown causes emerged in Wuhan, Hubei, China (Lu et al., 2020). Days later, Chinese health authorities confirmed that this group was associated with a new coronavirus [severe acute respiratory syndrome coronavirus 2 (SARS-CoV-2)] (Hui et al., 2020), known as coronavirus disease 2019 (COVID-19). On 30 January 2020, the Emergency Committee of the International Health Regulations of the World Health Organization declared the outbreak of COVID-19 a public health emergency of international interest (PHEIC) (World Health, 2020). In this regard, Spain, one of the most affected countries worldwide, declared the “state of alarm” for the entire national territory. Thus, displacement restrictions were imposed on citizens, indicating the confinement in their homes. People could only circulate in public spaces to carry out activities such as purchasing food, pharmaceuticals, or essential goods.

Previous studies showed that confinement has a negative impact on the general psychological health (Mihashi et al., 2009), which could lead to emotional disorders (Yoon et al., 2016) such as depression (Hull, 2005), stress (Digiovanni et al., 2004), and anxiety (Fancourt et al., 2020; Husky et al., 2020). In this regard, a key psychological variable to manage stressful situations (Rutter, 2007), resilience, which is defined as an individual's capacity to overcome adversity, is also affected during the confinement (Carriedo et al., 2020). Before the COVID-19 outbreak, some studies reported that regular physical exercise was a useful tool to reduce symptoms of depression and anxiety, increasing self-esteem and even a decrease in the idea of suicide (Vancampfort et al., 2018; Werneck et al., 2019), counteracting the effect of the confinement or isolation (Bonnet and Arand, 2001; Schneider et al., 2013). Thus, physical exercise was used during COVID-19 as a strategy to combat the psychological and physical consequences of the confinement, and its practice has been widely recommended during this period (Polero et al., 2021).

However, a recent systematic review did not find a consensus in the recommendations, the type, nor the intensities of the physical exercise during the confinement. Among all these recommendations, some studies recommended high-intensity interval training (HIIT) (Eirale et al., 2020; Narici et al., 2020) and others moderate-intensity training (MIT) (Chen et al., 2020; Fallon, 2020; Halabchi et al., 2020; Jiménez-Pavón et al., 2020) as an alternative for exercising during the confinement. HIIT is a high-intensity [greater than or equal to 85% of the heart rate maximum (HRmax)] interval training with short recovery periods (Weston et al., 2014), whereas MIT consisted of exercises between 70 and 85% HRmax. Previous evidence suggests that both HIIT (Martland et al., 2020) and MIT (Moholdt et al., 2009; Health and Human, 2018; Byrd et al., 2019) improve physical and psychological outcomes, anxiety, or depression, although adherence to physical exercise seems to be greater in HIIT (Heinrich et al., 2014).

Due to the lack of consensus and the relevant role of intensity in physical training (Macinnis and Gibala, 2017), it is needed to investigate, through randomized controlled trials, if HIIT or MIT has the same or different impacts on people's psychological health during the confinement situation. To date, there is no study comparing the effects of HIIT and moderate-intensity exercises (MIT) on the mental health of adult people during the COVID-19 confinement. Only one article analyzed the effects of physical training during the confinement (Vitale et al., 2020). This article studied the effects of home-based resistance training on physical fitness (measured by the chair-stand test). However, this article is not focused on the psychological health (which has another aspect that is dramatically affected by this situation) (Ammar et al., 2020). Therefore, the aim of the present randomized controlled trial was to evaluate the effects of two home-based exercise programs (one focused on HIIT and another on MIT) in the psychological health of adults during the COVID-19 confinement. As a primary objective, we analyzed the PRE-to-POST improvements (within and between groups) on depression after 6 weeks of home-based physical exercise. As secondary objectives, we analyze the PRE-to-POST improvements (within and between groups) on stress, anxiety, and resilience after 6 weeks of the COVID-19 confinement. We hypothesized that both home-based interventions (HIIT and MIT) would determine positive effects on the psychological health (i.e., depression, anxiety, stress, and resilience), being even greatest in the HIIT group. The rationale was that home-based exercise could be a useful and effective tool to maintain the psychological health during the COVID-19 confinement.

## Methods

### Study Design

The study is a randomized, single-blind controlled trial. Participants were divided into two groups: the MIT group, which underwent an aerobic exercise-based training program, and the HIIT group, which performed a HIIT exercise-based training program. The primary outcome, symptoms of depression [using the 13-item Beck Depression Inventory (BDI-13) (Beck et al., 1974)], and the secondary outcomes of stress [through the Perceived Stress Scale (PSS-10) (Cohen, 1988)], state of anxiety [using the State-Trait Anxiety Inventory (STAI-E) (Spielberger et al., 1971)], and resilience [through Connor-Davidson Resilience Scale (CD-RISC) (Campbell-Sills and Stein, 2007)] were assessed before and after 6 weeks of the intervention (the whole duration of strict confinement).

Some inclusion criteria were defined: (a) men or women aged between 18 and 65 years; (b) do not suffer from any musculoskeletal injury; (c) have been infected by COVID-19 or having symptoms of it; (d) do not practice other physical activities apart from prescribed by our research team during the duration of this trial; and (e) do not have any absolute contraindication for physical exercise practice. Participants who did not fulfill these criteria were not group-allocated.

The research ethics committee approved all the procedures of the University of Extremadura (approval number: 56/2020). Participants were informed of the procedures and gave their written consent prior to enrolment.

This trial was registered in the Australian New Zealand Clinical Trials Registry (ACTRN12620000482965), and the protocol was also published at https://www.anzctr.org.au/ACTRN12620000482965.aspx.

### Participant Screening

Once the Spanish government declared the “state of alarm” (March 2020) for the entire national territory and that people must be confined in their homes (people could only circulate in public spaces to carry out activities such as purchasing food, pharmaceuticals, or essential goods), two of the researchers announced in social media (Instagram and Facebook) for volunteers who wanted to participate in a home-based physical activity program during the COVID-19 confinement. Interested people contacted by direct message the researchers. A total of 76 participants contacted the researchers and met the inclusion criteria. Any of the participants were professional sportsmen or sportswomen. The majority of the participants included in this study manifested a low level of physical activity (55.23%). In contrast, moderate and high levels of physical activity were detected on 19.40 and 23.37% of the participants, respectively, according to the data extracted from the International Physical Activity Questionnaire (IPAQ) (Craig et al., 2003) when asked about their physical activity habits before the COVID-19 home confinement. Further characteristics of the sample can be checked in Table 1.

**Table 1:** Descriptive of the sample.

	**Total Mean (SD)**	**HIIT group Mean (SD)**	**MIT group Mean (SD)**	***p*-value**
Sample size (*n*)	67	36	31	
Age (years)	26.13 (7.17)	25.22 (5.23)	27.19 (8.88)	0.668
Sex [women (men)]	45 (22)	21 (15)	24 (7)	0.097
STAI-E	46.64 (9.90)	47.33 (9.31)	45.84 (10.62)	0.541
BDI-13	4.64 (3.87)	5 (4.10)	4.23 (3.62)	0.531
PSS-10	18.52 (6.21)	19 (6.51)	18 (5.90)	0.650
CD-RISC10	30.28 (6.26)	31.14 (5.2)	29.29 (7.27)	0.371
**IPAQ level of physical activity**				
Low^a^	37 (55.23%)	19 (52.78%)	18 (58.06%)	0.547
Moderate^b^	13 (19.40%)	6 (16.67%)	7 (22.58%)	
High^c^	17 (25.37%)	11 (30.57%)	6 (19.35%)	

*BDI-13, 13-item Beck Depression Inventory; CD-RISC10, Connor-Davidson Resilience Scale; HIIT, high-intensity interval training; IPAQ, International Physical Activity Questionnaire; MIT, moderate-intensity training; PSS-10, Perceived Stress Scale; SD, standard deviation; STAI, State-Trait Anxiety Inventory*.

a *The participants did not meet any of the criteria for either moderate or high levels of physical activity according to the IPAQ*.

b *Participant performed some activity equivalent to 0.5 h of at least moderate intensity on most days*.

c *Participant performed ~1 h of activity per day or more of at least a moderate-intensity activity level*.

Participants were randomly allocated into the two groups (HIIT or MIT) by a technician using random numbers. This researcher did not take part in the acquisition, intervention, or data analysis. Another technician performed the intervention, and another collected the daily adherence as well as initial and final tests. Furthermore, participants were also blinded to group allocation since physical training sessions were individually sent.

A Google Form was created to collect sociodemographic data (such as age, sex, or physical activity habits) and primary and secondary outcomes (symptoms of depression, state of anxiety, perceived stress, and resilience). These online questionnaires were administrated at two pointlines: before and after the home-based interventions.

### Training Protocol

The two groups (HIIT and MIT) had to complete the same physical exercise volume, 40 min per session (6 days per week) during the confinement period (6 weeks). A kinesiologist with more than 5 years of experience provided a video session uploaded to YouTube. A WhatsApp message was individually sent to each participant with a session link that they had to complete.

- MIT group. This group had to perform a home-based intervention based on aerobic exercises. Each session had the following:

1. A warm-up (10 min): joint mobility exercises.
2. Main part: three to four blocks of 6–8 min with 1- to 2-min hydration pause between them (i.e., boxing squats, jumping jacks, skipping, or skaters). All the exercises were performed with their own weight or with an extra weight when needed, using small (≈500 g) or large (≈1,500 g) water bottles, at 4–6 of their maximum perceived effort, which corresponds to a 70–85% HRmax (Borg, 1970; Buceta, 1998; Edwards and Brown, 2003). Participants were informed about the rate of perceived exertion (RPE) on a 0–10 scale and were encouraged to maintain the required intensity during the sessions (Borg, 1970). The exercise description, sets, duration, repetitions resting time, exercise progression, and load are detailed in Table 2.
3. Cooldown: For the main body muscles (mainly back, neck and upper and lower limbs), which were involved during the training session, static stretching exercises were conducted, lasting 30–40 s.

**Table 2:** Description of the MIT intervention exercises performed during the central part of the home-based intervention.

**Exercise type**	**Number of sets**	**Duration**	**Resting time**	**Exercise progression**	**Intensity**	**Load**
**Boxing squats** 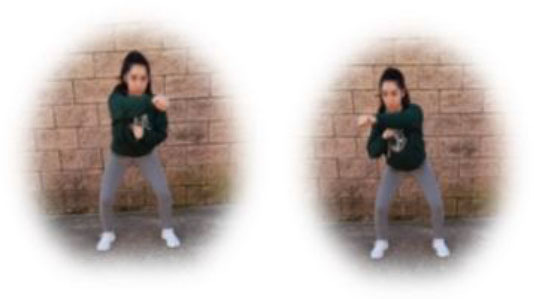	3	6–8 min	1–2 min	Increase of sets (up to *n* = 4) and/or execution time (up to 8 min) and/or decrease resting time (up to 1 min)	4–6 of RPE	Body weight
**Jumping jacks** 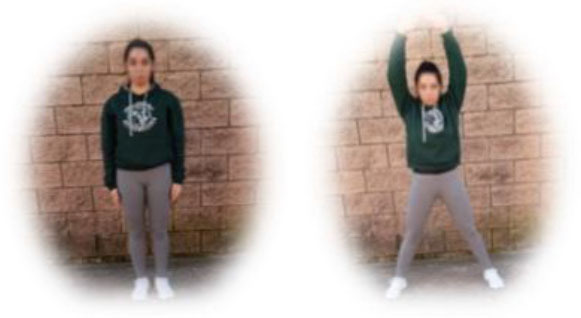				Increase of sets (up to *n* = 4) and/or execution time (up to 8 min) and/or decrease resting time (up to 1 min)	4–6 of RPE	Body weight
**Skipping** 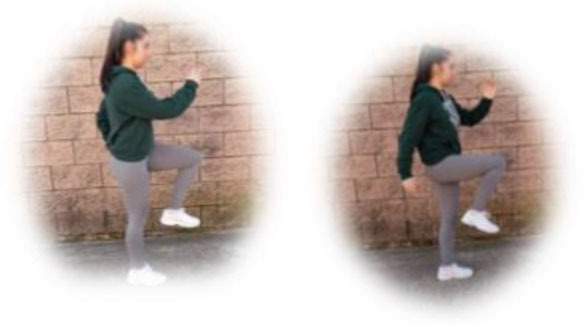				Increase of sets (up to *n* = 4) and/or execution time (up to 8 min) and/or decrease resting time (up to 1 min)	4–6 of RPE	Body weight
**Skaters** 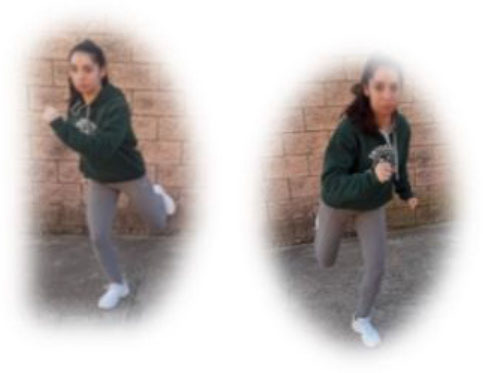				Increase of sets (up to *n* = 4) and/or execution time (up to 8 min) and/or decrease resting time (up to 1 min)	4–6 of RPE	Body weight
**Climbers** 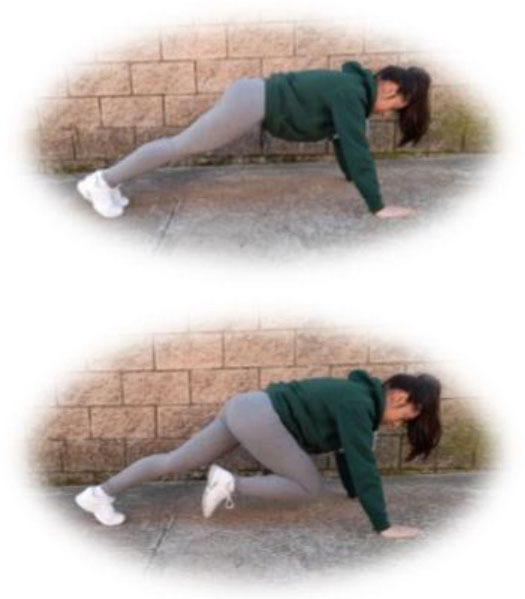				Increase of sets (up to *n* = 4) and/or execution time (up to 8 min) and/or decrease resting time (up to 1 min)	4–6 of RPE	Body weight

*MIT, moderate-intensity training; RPE, rate of perceived exertion*.

- HIIT group. This group had to perform a home-based intervention based on HIIT exercises. Each session had the following:

1. A warm-up (10 min): joint mobility exercises.
2. Main part: core, arm, and leg exercises, with 10–12 sets of 30–90 s with 15–60 s of rest between sets (i.e., push-ups, squats, splits, or deadlifts). All the exercises will be performed with their own weight at 7–9 of their maximum perceived effort, which corresponds to an 85–95% HRmax (Borg, 1970; Buceta, 1998; Edwards and Brown, 2003). Participants were informed about the RPE on a 0–10 scale and were encouraged to maintain the required intensity during the sessions (Borg, 1970). The exercise description, sets, duration, repetitions resting time, exercise progression, and load are detailed in Table 3.
3. Cooldown: For the main body muscles (mainly back, neck, and upper and lower limbs), which were involved during the training session, static stretching exercises were conducted, lasting 30–40 s.

**Table 3:** Description of the HIIT intervention exercises performed during the central part of the home-based intervention.

**Exercise type**	**Number of sets**	**Duration**	**Resting time**	**Exercise progression**	**Intensity**	**Load**
**Pushup** 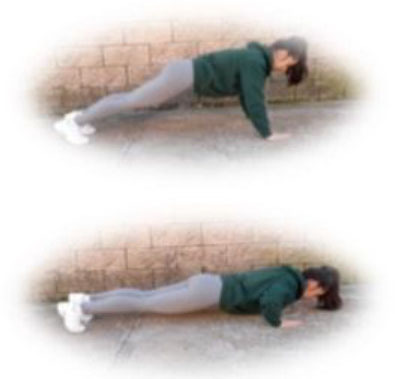	3	30–90 s	15–60 s	Increase of sets (up to *n* = 4) and/or execution time (up to 90 s) and/or decrease resting time (up to 15 s) and/or start kneeling on the extending arms into a high plank position	6–9 of RPE	Body weight
**Squat** 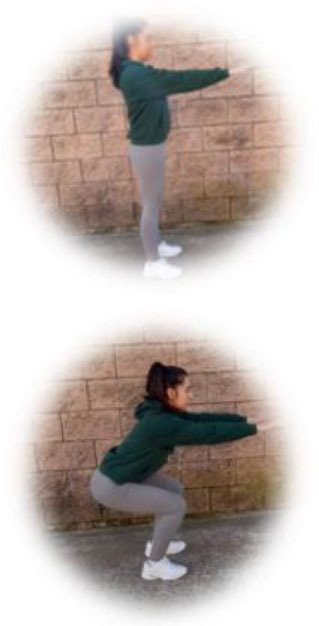	3	30–90 s	15–60 s	Increase of sets (up to *n* = 4) and/or execution time (up to 90 s) and/or decrease resting time (up to 15 s) and/or use of one bottle as extra weights	6–9 of RPE	Body weight or bottles
**Split** 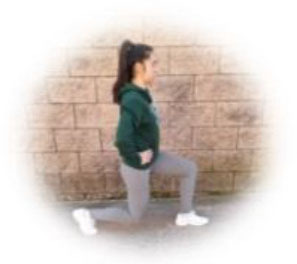	3	30–90 s	15–60 s	Increase of sets (up to *n* = 4) and/or execution time (up to 90 s) and/or decrease resting time (up to 15 s) and/or use of two bottles as extra weights	6–9 of RPE	Body weight or bottles
**Dead lift** 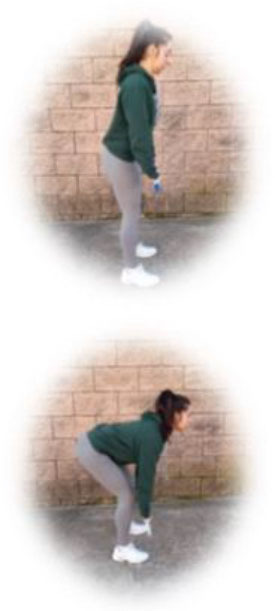	3	30–90 s	15–60 s	Increase of sets (up to *n* = 4) and/or execution time (up to 90 s) and/or decrease resting time (up to 15 s) and/or use two bottles as extra weights	6–9 of RPE	Body weight or bottles
**Plank** 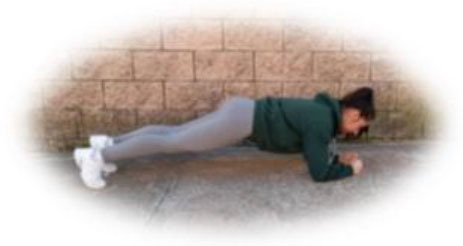	3	30–90 s	15–60 s	Increase of sets (up to *n* = 4) and/or execution time (up to 90 s) and/or decrease resting time (up to 15 s)	6–9 of RPE	Body weight

*HIIT, high-intensity interval training; RPE, rate of perceived exertion*.

Training adherence was controlled daily by one of the researchers. All participants had to send a WhatsApp message when they ended the training session. The minimum acceptable adherence to interventions was 75%. Participants who did not reach this percentage were considered as having discontinued the intervention.

### Psychological Profile Monitoring

Participants completed the following questionnaires before and after the intervention: Symptoms of depression were assessed using the Spanish version of the BDI-13 (Beck et al., 1974; Bobes et al., 2004), showing a reliability of 0.83 (Sanz and Vázquez, 1998). It consists of 13 items with four possible response options ranging from 0 to 3 points: 0 = I don't feel sad, 1 = I feel sad, 2 = I feel sad continuously and I can't stop being sad, and 3 = I feel so sad that I can't bear it. The total score varies from 0 to 39 points, considering absent depression from 0 to 4 points, mild depression from 5 to 7 points, moderate depression from 8 to 15 points, and severe depression at more than 15 points (Beck et al., 1974; Collet and Cottraux, 1986).

State of anxiety, in its Spanish version, was assessed through the STAI-E (Spielberger et al., 1971; Buela-Casal et al., 1982), showing a Cronbach's alpha of 0.94 (Guillén-Riquelme and Buela-Casal, 2011). It consists of 20 items; and when responding, the subjects report their state of anxiety at that time through their responses, which can be 1 = nothing, 2 = little, 3 = a lot, and 4 = a lot. The score range for the test is 20–80, indicating a higher level of anxiety with a higher score (Spielberger et al., 1971).

The Spanish version of the stress scale (PSS-10) was used to measure the stress, showing a Cronbach's alpha = 0.82 and a test–retest, r = 0.77 (Remor, 2006). This scale consists of 10 items scored as follows: 0 = never, 1 = almost never, 2 = from time to time, 3 = often, and 4 = very often. The total score is between 0 and 40 points, considering those with the highest score with the highest stress (Cohen, 1988).

Resilience was evaluated using the Spanish version of the CD-RISC (Campbell-Sills and Stein, 2007; Soler Sánchez et al., 2016), which has shown a Cronbach's alpha = 0.87. The short version of this scale was administered to assess the resilience to problems. It consists of 10 items with five answer options: 0 = not at all, 1 = rarely, 2 = sometimes, 3 = often, and 4 = almost always. The scale range goes from 0 to 40. The higher the score, the more resilience (Campbell-Sills and Stein, 2007).

In order to characterize the participants in their level of physical activity prior to the COVID-19 home confinement, the Spanish version of the IPAQ was administered (Craig et al., 2003; Roman-Viñas et al., 2010). Participants were encouraged to think about the physical activity behaviors before the start of the home confinement. Therefore, each participant's level of physical activity was calculated: high, moderate, or low level. High level was achieved by participants who performed vigorous-intensity activity on at least 3 days, achieving a minimum total physical activity of at least 1,500 metabolic equivalent of task (MET) minutes a week or performed seven or more days of any combination of walking, and moderate-intensity or vigorous-intensity activities, achieving a minimum total physical activity of at least 3,000 MET minutes a week. Moderate level was achieved by participants engaged in three or more days of vigorous-intensity activity and/or walking of at least 30 min per day; or five or more days of moderate-intensity activity and/or walking of at least 30 min per day; or five or more days of any combination of walking, and moderate-intensity or vigorous-intensity activities, achieving a minimum total physical activity of at least 600 MET minutes a week. Low levels of physical activity were achieved by participants who did not fulfill the criteria for either moderate or high physical activity levels (Craig et al., 2003; Committee, 2005). The Spanish version of the IPAQ showed a good reliability coefficient for total physical activity (r = 0.82, *p* < 0.05), vigorous activity (r = 0.79, *p* < 0.05), moderate activity (r = 0.83, *p* < 0.05), and time spent walking (r = 0.73, *p* < 0.05) (Roman-Viñas et al., 2010).

### Data Analysis

The STAI was employed in order to estimate the sample size using the PASS software (version 11; NCSS, LLC, Kaysville, Utah, USA) (Hintze, 2008). Taking into account the data from a previous research (Mailey et al., 2010), 37 participants per group were estimated in order to detect differences (α value 0.05 and 99% of power) in a repeated measures design.

The SPSS statistical package (version 20.0; SPSS, Inc., Chicago, Ill.) was used to analyze the data. Non-parametric tests were performed based on the results of the Shapiro–Wilk and Kolmogorov–Smirnov tests.

Data from the initial 67 participants were used to perform the intention-to-treat analysis using multiple imputations (MIs) of missing values, following the guidelines of Sterne et al. (2009). Thus, the missing data were classified as missing at random.

Mann–Whitney *U*-test was used to study differences at baseline in age, gender, and scores on the STAI-E, BDI-13, PSS-10, and CD-RISC10 questionnaires. A chi-squared test was used to study differences between groups in the physical activity level extracted from the IPAQ.

The difference between the post and the pre was also calculated for each variable. These differences were used to study the between-group effects of the intervention by the Mann–Whitney *U*-test in the different variables. Within-group effects were explored using the Wilcoxon test between pre-test and post-test.

Effect sizes [r] were calculated for the non-parametric tests, which is classified as follows: 0.5 is a large effect, 0.3 is a medium effect, and 0.1 is a small effect (Fritz et al., 2012; Coolican, 2018).

## Results

**Figure 1:**
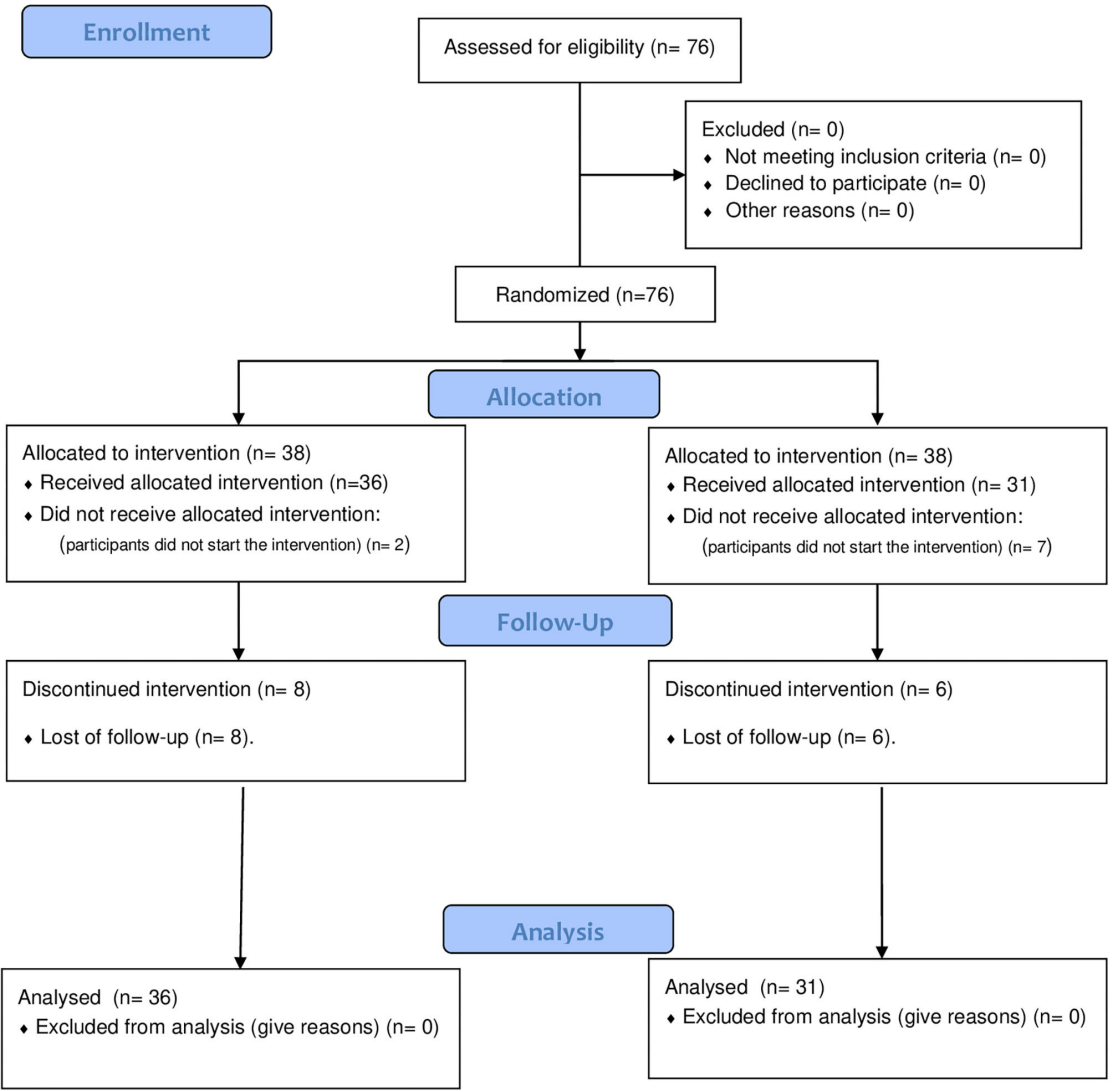
Flow chart of participants.

The flowchart is shown in Figure 1. A total of 76 were randomly allocated in the two groups. However, seven participants from the MIT group and two participants from the HIIT group did not start the intervention. Therefore, 67 adults started the intervention (36 from the HIIT and 31 from MIT groups). However, during the home-based intervention, eight participants from the HIIT group and six participants from the MIT group dropped out of the intervention (due to demotivation with the exercise interventions). Nevertheless, intention-to-treat analyses were applied to the data of these participants.

Baseline characteristics are reported in Table 1. No significant differences (*p* > 0.05) were observed between the HIIT and MIT groups in age, gender, and the total score of stress (PSS-10), depression (BDI-13), anxiety (STAI-E), resilience (CD-RISC10), or physical activity levels (IPAQ) at baseline.

Table 4 shows the effect of HIIT and MIT on anxiety, depression, stress, and resilience. Wilcoxon signed-rank tests showed significant effects in both groups for all the studied variables: anxiety (HIIT *p* = 0.014; MIT *p* = 0.034), depression (HIIT *p* ≤ 0.001; MIT *p* = 0.016), stress (HIIT *p* ≤ 0.001; MIT *p* = 0.013), and resilience (HIIT *p* = 0.049; MIT *p* = 0.004). Based on the effect size [r], the effects could be classified as medium/large in all the variables.

**Table 4:** Effects of 6 weeks of HIIT and MIT on anxiety, depression, stress, and resilience after applying intention-to treat analysis.

**Variables**				**Within-group analyses**			**Between groups analyses**		
		**Pre Mean (SD)**	**Post Mean (SD)**	***Z***	***p*-value**	**Effect size**	***Z***	***p*-value**	**Effect size**
**STAI-E**									
Anxiety	HIIT	47.33 (9.31)	42.45 (10.76)	−2.449	0.014	0.408	−0.673	0.501	0.082
	MIT	45.84 (10.62)	42.72 (11.75)	−2.120	0.034	0.381			
**BDI-13**									
Depression	HIIT	5 (4.10)	2.5 (2.74)	−4.161	<0.001	0.693	−1.998	0.046	0.244
	MIT	4.23 (3.62)	2.61 (2.68)	−2.419	0.016	0.434			
**PSS-10**									
Stress	HIIT	19 (6.51)	14.83 (6.92)	−4.184	<0.001	0.697	−0.327	0.743	0.039
	MIT	18 (5.90)	14.65 (6.62)	−2.481	0.013	0.445			
**CD-RISC10**									
Resilience	HIIT	31.14 (5.2)	32.04 (5.93)	−1.961	0.049	0.327	−1.028	0.304	0.125
	MIT	29.29 (7.27)	32.06 (6.16)	−2.861	0.004	0.514			

*BDI-13, 13-item Beck Depression Inventory; CD-RISC10, Connor-Davidson Resilience Scale; HIIT, high-intensity interval training; MIT, moderate-intensity training; PSS-10, Perceived Stress Scale; SD, standard deviation; STAI, State-Trait Anxiety Inventory*.

Mann–Whitney *U*-tests showed that the HIIT intervention significantly reduced the depression symptoms more than the MIT intervention [*p* = 0.046; [r] = 0.244] with an effect size that can be classified as medium. However, significant differences were not observed in anxiety (*p* = 0.501), stress (*p* = 0.743), or resilience (*p* = 0.304).

Considering the 75% attendance criterion, the final adherence was 77.78% and 80.64% for the HIIT and MIT, respectively. No side effects derived from any of the interventions were detected.

## Discussion

This study aimed to compare the effects of two intervention programs (HIIT and MIT) on anxiety, depression, stress, and resilience during the confinement caused by COVID-19 in healthy adults. Results showed that HIIT and MIT reduced stress, anxiety, and depression as well as increase resilience. Moreover, the improvements obtained in the HIIT group seem to be greater than those of the MIT group in depression.

The COVID-19 home confinement increased the level of sedentarism among the general population (Fuentes-García et al., 2020; Narici et al., 2020). This, linked to a high uncertainty and stress (Lin et al., 2020), led to a significant decrease in the psychological health (Ammar et al., 2020). Previous studies indicated that the COVID-19 home confinement had a significant impact on stress, anxiety, and depression (Fancourt et al., 2020; Husky et al., 2020). Thus, due to the positive effects of physical exercise on the psychological and physical health, it was highly recommended during this the COVID-19 home confinement (Polero et al., 2021). Our results confirm that regular home-based exercise could fight the negative impact of the COVID-19 confinement on the psychological health, decreasing stress, anxiety, and symptoms of depression while increasing resilience.

Furthermore, previous studies have focused on the importance of physical exercise during the COVID-19 confinement (Chtourou et al., 2020; Jiménez-Pavón et al., 2020). In this regard, our results are consistent with a previous cross-sectional study, which reported that those people who were not enrolled in physical exercise during the COVID-19 confinement showed a higher level of stress, anxiety, and depression (Silva et al., 2020). Moreover, same as this study, another one conducted a randomized controlled trial focused on the effects of a home-based resistance training on the physical fitness of older adults. Results highlighted the importance of home-based physical training to reduce the impact of the COVID-19 confinement on the physical fitness. However, this study was focused on the physical dimension, whereas our study showed, for the first time, that home-based intervention could improve the relevant psychological outcomes such as stress, anxiety, or depression.

Importantly, the HIIT and MIT interventions improved resilience (ability to recover and maintain adaptive behavior after a stressful event) (Garmezy, 1991). This is relevant since resilience is quite important to face uncertain situations under stress, such as the COVID-19 pandemic (Bryce et al., 2020; Nitschke et al., 2020). Thus, a previous study claimed for factors that could promote resilience during this period (Vinkers et al., 2020). Therefore, both HIIT and MIT home-based physical exercise interventions could be used to improve resilience during the COVID-19 home confinement. This idea is supported by a previous study, which showed that those who were enrolled in vigorous physical exercise obtained higher values of resilience during the confinement (Carriedo et al., 2020). Nevertheless, we did not observe statistically significant differences between HIIT and MIT. However, differences between groups were found between HIIT and MIT in the symptoms of depression (with significantly lower values in the HIIT group after the home-based intervention than in the MIT group). Hypothetically, this could be explained by the work done per unit time, which is greater in the HIIT than in the MIT. This is in line with a previous study (Luo et al., 2019) where the HIIT group obtained a greater effect on depression than did the MIT group. In this regard, HIIT training has been related to endogenous opioid activation, contributing to greater stress relief (Schwarz and Kindermann, 1992) as well as mood and depression (Firth et al., 2015; Schuch et al., 2016).

Adherence to physical activity is one of the most relevant challenges in public health (Dishman, 1988; Matthews et al., 2008) since it is crucial for benefits maintenance. Previous studies have reported that the HIIT appears to be more enjoyable than MIT, since short recovery periods can provide relief from active exercise, in contrast to continuous exercise (Tjønna et al., 2008; Bartlett et al., 2011). A previous systematic review reported that the adherence to HIIT interventions exceeds the 80%. However, in our intervention, adherence was quite similar in both MIT (80.64%) and HIIT (77.78%) protocols. Furthermore, another randomized controlled trial during the COVID-19 outbreak (using home-based resistance training) reported an adherence of 84.8% (with the same percentage of attendance–75%). However, the sample size of this randomized controlled trial was lower than in ours, allocating in the exercise group nine adults (four dropped out) and five in the control group (one dropped out).

This study has some limitations that should be acknowledged. First, the confinement did not allow us to control the intensity through heart rate monitors. Second, results cannot be extrapolated to special populations, since healthy people participated in this study. Moreover, future studies should incorporate physiological outcomes in order to confirm our results. Third, in the present study, gender differences in the effectiveness of the physical exercise, which can affect stress, depression, or anxiety, were not explored. Nevertheless, our study has some strong points that should be also highlighted. In this regard, only one previous article conducted a randomized controlled trial to study the effects of home-based interventions (Vitale et al., 2020), and this is the first that studies the effectiveness of two interventions (HIIT and MIT) on symptoms of depression, anxiety, stress, and resilience. Moreover, the intervention was conducted under an ecological context, with any sports material and associated cost, and therefore anyone could participate. In contrast, other physical exercise alternatives emerged during the COVID-19 home confinement, such as exergames (Viana and De Lira, 2020). However, in contrast with our intervention, the associated cost of these interventions is higher than in home-based physical exercise interventions. Considering all these strong points, this research could be considered as timely and quite relevant since these accessible home-based physical exercise interventions could be used against the negative impacts of COVID-19 on both physical and mental health.

## Conclusions

HIIT and MIT decreased anxiety, stress, and depression as well as increased resilience during the COVID-19 confinement. In addition, the HIIT intervention seemed to be more beneficial to reduce depression than the MIT intervention. Thus, HIIT or MIT home-based sessions should regularly be applied (under professional supervision) in order to maintain the person physically active during the COVID-19 home confinement. This would lead to decrease anxiety, stress, and symptoms of depression as well as to increase resilience. Therefore, results are timely and quite relevant in this situation in which people are isolated and where physical activity has a crucial role in maintaining both mental and physical health.

## Data Availability Statement

The raw data supporting the conclusions of this article will be made available by the authors, without undue reservation.

## Ethics Statement

The studies involving human participants were reviewed and approved by University of Extremadura Research Ethics committee (56/2020). The patients/participants provided their written informed consent to participate in this study.

## Author Contributions

SV, YB-M, and DC-M conceived and designed the study. YB-M and JS-G assisted with recruitment of participants. JF-G and SV conducted the statistical analysis and interpretation of data. YB-M drafted the manuscript with input from SV, DC-M, JS-G, and JF-G. All authors have read and approved the final manuscript.

## Conflict of Interest

The authors declare that the research was conducted in the absence of any commercial or financial relationships that could be construed as a potential conflict of interest.
